# Sensitizing thermochemotherapy with a PARP1-inhibitor

**DOI:** 10.18632/oncotarget.11422

**Published:** 2016-08-19

**Authors:** Arlene L Oei, Lianne E.M Vriend, Caspar M. van Leeuwen, Hans M Rodermond, Rosemarie ten Cate, Anneke M Westermann, Lukas J.A. Stalpers, Johannes Crezee, Roland Kanaar, Kok H. Petra, Przemek M Krawczyk, Nicolaas A.P. Franken

**Affiliations:** ^1^ Laboratory for Experimental Oncology and Radiobiology (LEXOR), Center for Experimental Molecular Medicine (CEMM), Academic Medical Center (AMC), 1100 DE, Amsterdam, The Netherlands; ^2^ Department of Radiotherapy, Academic Medical Center (AMC), 1100 DE, Amsterdam, The Netherlands; ^3^ Department of Cell Biology and Histology, Academic Medical Center (AMC), 1100 DE, Amsterdam, The Netherlands; ^4^ Department of Medical Oncology, Academic Medical Center (AMC), 1100 DE, Amsterdam, The Netherlands; ^5^ Department of Molecular Genetics, Cancer Genomics Center Netherlands, The Netherlands; ^6^ Department of Radiation Oncology, Erasmus University Rotterdam (EUR), 3000 DR Rotterdam, The Netherlands

**Keywords:** PARP1-inhibitor, hyperthermia, synthetic lethality, cDDP, RAD51

## Abstract

Cis-diamminedichloroplatinum(II) (cisplatin, cDDP) is an effective chemotherapeutic agent that induces DNA double strand breaks (DSBs), primarily in replicating cells. Generally, such DSBs can be repaired by the classical or backup non-homologous end joining (c-NHEJ/b-NHEJ) or homologous recombination (HR). Therefore, inhibiting these pathways in cancer cells should enhance the efficiency of cDDP treatments. Indeed, inhibition of HR by hyperthermia (HT) sensitizes cancer cells to cDDP and in the Netherlands this combination is a standard treatment option for recurrent cervical cancer after previous radiotherapy. Additionally, cDDP has been demonstrated to disrupt c-NHEJ, which likely further increases the treatment efficacy. However, if one of these pathways is blocked, DSB repair functions can be sustained by the Poly-(ADP-ribose)-polymerase1 (PARP1)-dependent b-NHEJ. Therefore, disabling b-NHEJ should, in principle, further inhibit the repair of cDDP-induced DNA lesions and enhance the toxicity of thermochemotherapy. To explore this hypothesis, we treated a panel of cancer cell lines with HT, cDDP and a PARP1-*i* and measured various end-point relevant in cancer treatment. Our results demonstrate that PARP1-*i* does not considerably increase the efficacy of HT combined with standard, commonly used cDDP concentrations. However, in the presence of a PARP1-*i*, ten-fold lower concentration of cDDP can be used to induce similar cytotoxic effects. PARP1 inhibition may thus permit a substantial lowering of cDDP concentrations without diminishing treatment efficacy, potentially reducing systemic side effects.

## INTRODUCTION

Cisplatin (cis-diamminedichloroplatinum(II), cDDP), is one of the most potent and widely used chemotherapeutic agents. cDDP has been successfully applied in treating many different tumor types, including head and neck, lung, testis, ovarian, cervix and breast [1, 2]. cDDP induces formation of intra- and interstrand crosslinks, which may induce DNA single and double strand breaks (SSBs and DSBs) in replicating cells [3, 4]. The accumulation of unrepaired DNA lesions, particularly DSBs, can lead to cell death. In mammalian cells, DSBs are repaired by mainly two major pathways: the classical non-homologous end joining (c-NHEJ) and the homologous recombination (HR). HR is mainly active in S- and G_2_-phase, ensures accurate repair by using the undamaged sister chromatid as a template and involves, among others, BRCA2 and RAD51 [5]. Mild hyperthermia (HT), 1 h at 41–42.5°C, can disrupt this pathway temporarily by inducing degradation of BRCA2 and preventing the recruitment of RAD51 to DSBs [6]. c-NHEJ is active in all cell cycle phases, rejoins the break ends without the requirement for homologous template and is therefore considered to be more error prone [7–9]. Besides inducing DNA breaks, cDDP has also been shown to disrupt c-NHEJ [10–13].

The combination of cDDP and HT has been shown to be an effective clinical modality and is the standard treatment for previously irradiated patients with recurrent cervical cancer in the Netherlands [14]. Nevertheless, commonly used dose of cDDP can be very toxic for a substantial percentage of patients due to sometimes irreversible neural toxicity (hearing loss, numbness and tingling of the extremities) and kidney failure [15, 16]. Kidney failure is particularly dreaded in women with advanced cervical cancer in whom kidney function is already impaired by renal obstruction due to the tumor. Therefore, a large number of patients would benefit it the required dose of cDDP could be reduced without compromising tumor control.

Tumor resistance to cDDP plus HT may not only be explained by an insufficient cDDP dose, but also by the existence of a third DNA-repair pathway, so-called back-up NHEJ (b-NHEJ), which becomes active when either or both c-NHEJ and HR are impaired [17]. Poly-(ADP-ribose)-polymerase1 (PARP1) is essential for functioning of b-NHEJ [18, 19], and at least partly explains the accumulation of DSBs. Additional benefits of this approach may come from the observations that PARP1 plays a crucial role in regulating replication fork progression. Collapsed replication forks caused by a PARP1-*i* are converted to DSBs and require HR for repair [20]. In HR deficient cells, including cells harboring inactivation mutations in BRCA1 or BRCA2 and cells suffering from a HT-induced BRCA2 degradation, such lesions become highly cytotoxic in what can be considered a form of synthetic lethality [6, 20–24]. Importantly, PARP1 inhibitors have already been in multiple clinical trials in BRCA negative breast and ovarian cancers, and generally show favorable clinical profile [25, 26].

Here we set out to test, *in vitro*, whether the efficacy of cDDP+HT modality can benefit from the inhibition of PARP1. We focused on two interrelated aspects of such therapeutic strategy. First, we investigated whether PARP1-*i* can enhance the cytotoxicity of the standard cDDP+HT regimen. Second, we asked whether addition of PARP1-*i* can allow significant reduction of the overall cDDP dose, while maintaining the cytotoxic potential of the treatment. This is a clinically relevant question, particularly in the case that inhibition of PARP1 does not significantly alter the efficacy of HT when combined with standard cDDP doses, due to relatively high cytotoxicity of the two modality approach. Given that the concentrations of cDDP in necrotic or poorly vascularized tumor areas are likely low, reducing the cDDP dose required for efficient cell killing by co-administering PARP1-*i* may allow maintaining local tumor control while limiting the systemic side effects associated with standard cDDP concentrations.

## RESULTS

### Mild hyperthermia induces cell cycle arrest, apoptosis and inhibits homologous recombination

**Figure 1:**
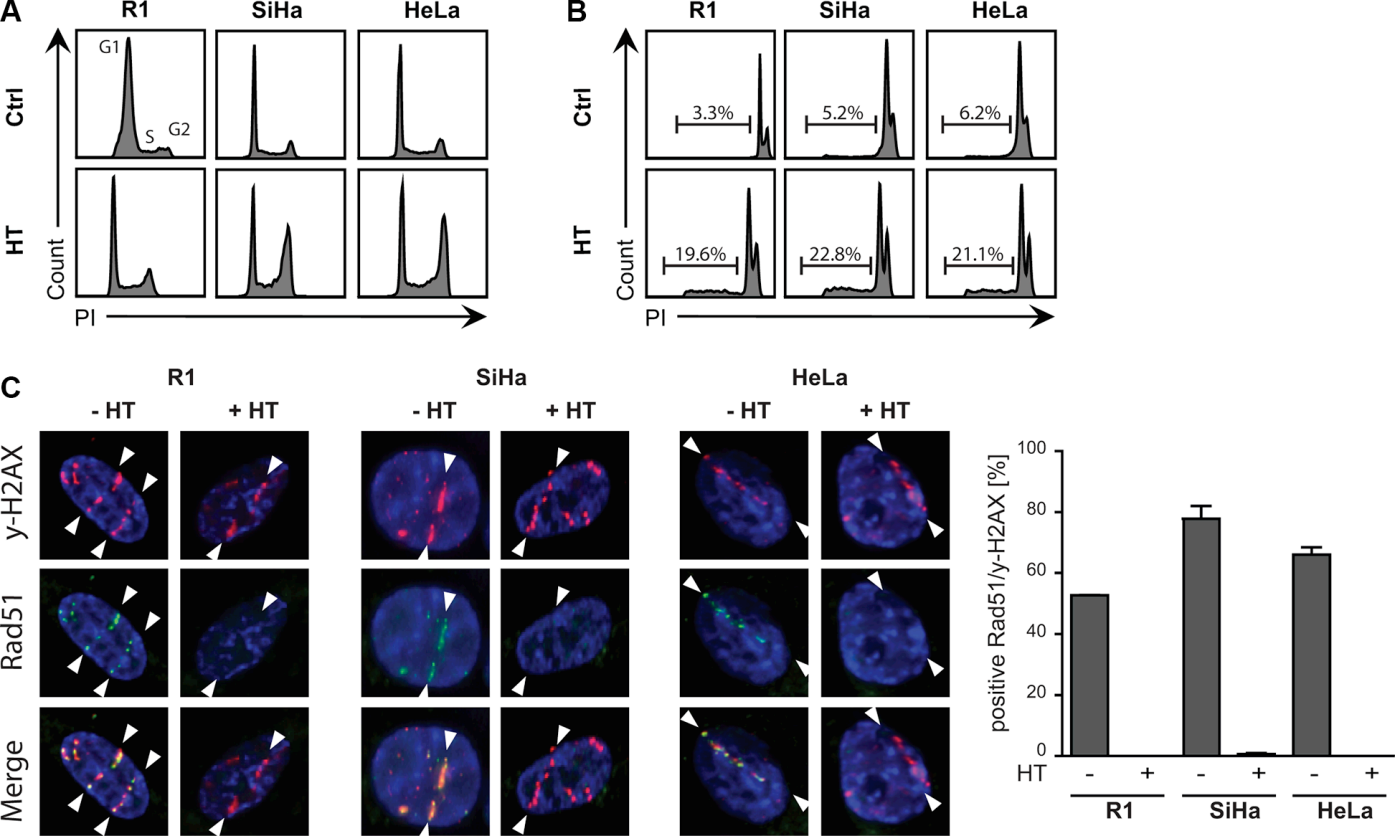
Sensitivity of cells to hyperthermia (**A**) Cell cycle analysis were determined via FACS analysis after BrdU incorporation. A G2-arrest is observed after HT treatment. (**B**) Apoptosis levels were studied using the Nicoletti assay. HT induced apoptosis in all cell lines. (**C**). Representative pictures of co-localization of γ-H2AX and RAD51 foci on α-irradiation tracks in untreated cells and after HT treatment. RAD51 is no longer detected 30 min after HT, indicating that HR is not active. The bar graph with the standard error of the mean shows the mean of at least three independent experiments. For each condition more than 300 cells were analyzed.

To determine the effect of HT on R1, SiHa and HeLa cells, we first measured changes in cell cycle distribution and induction of apoptosis. In the cell cycle analysis (Figure 1A) a G2-arrest was observed 16 h after treating cells for 1 h with 42**°**C. This effect was moderate for R1 cells and more pronounced for SiHa and HeLa cells. Flow cytometric analysis of DNA content showed a 20% increase in apoptosis for all cell lines (Figure 1B). Next, We measured the effects of HT on HR activity by quantifying accumulation of HR factor RAD51 on alpha-particle induced DSBs. Consistently with previously published results, HT treatment temporarily abrogated accumulation of RAD51 on DSB sites in all cell lines (Figure 1C), confirming inactivation of HR.

### PARP1-*i* sensitizes cells moderately to combinational treatment of cDDP with hyperthermia

**Figure 2:**
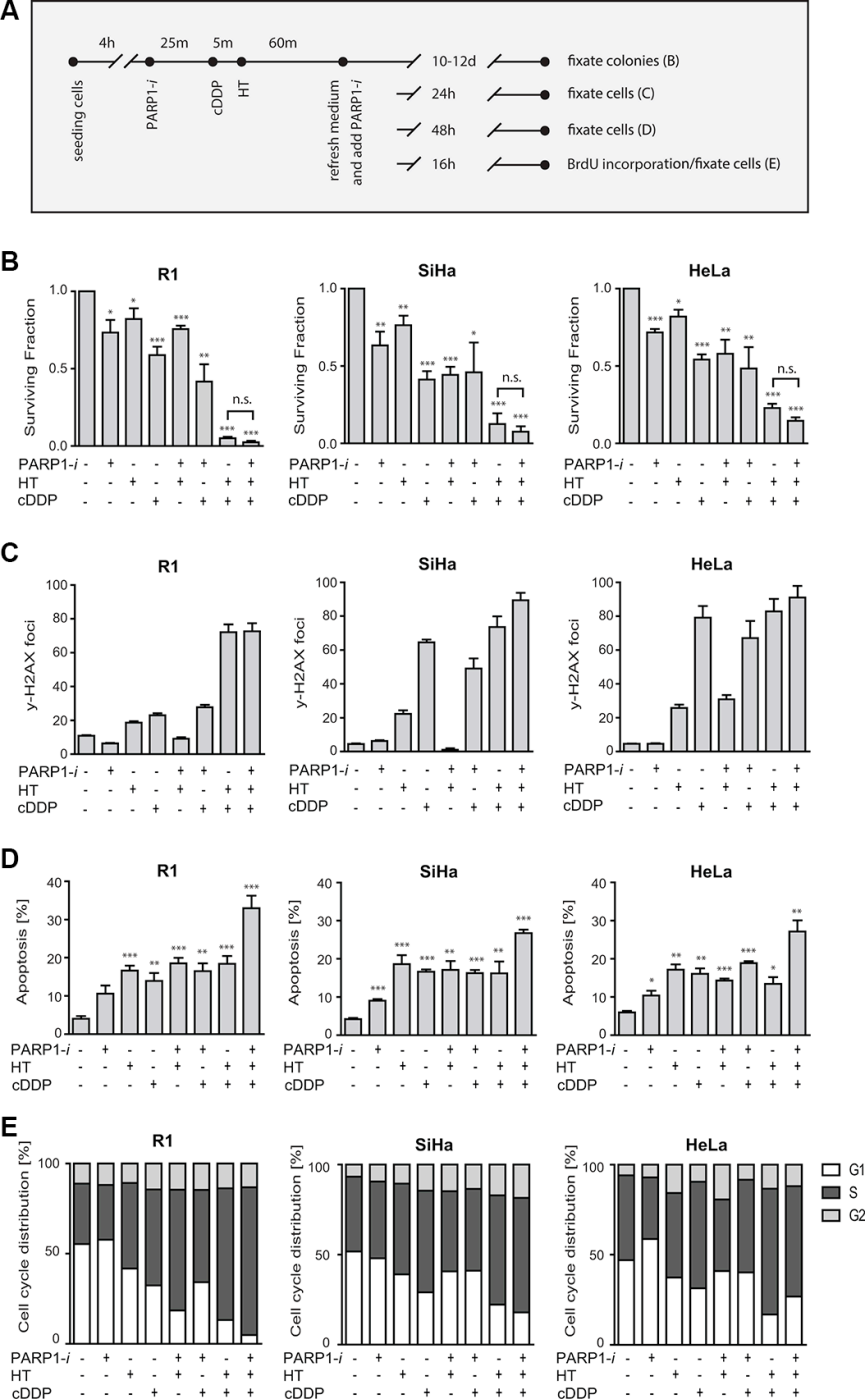
Effects of PARP1-*i* (100 μM NU1025/continuously), HT (42°C/1 h), cDDP (5 μM/1 h) (**A**) Overview of treatment schemes for different experiments represented in B–E. (**B**) Clonogenic assays were conducted in order to study the cell survival after 10–12 days post-treatment. No significant differences were found between HT+cDDP and cDDP + HT + PARP1-*i*. (R1: *p* = 0.10, SiHa: *p* = 0.12, HeLa: *p* = 0.10). (**C**) DNA DSBs were analyzed using the γ-H2AX assay. The induction of DSBs in SiHa and HeLa after cDDP treatment is 3-4 times higher than in R1 cells. Nonetheless, all three cells lines show around 70–80 DSBs after cDDP+HT or cDDP+HT+PARP1-*i*. (**D**) The Nicoletti assay was performed to observe apoptosis levels after different treatments. The triple combination treatment gave the highest levels of apoptosis. (**E**) Cell cycle was determined via FACS analysis after BrdU incorporation. cDDP+HT and cDDP+HT+PARP1-*i* show a slight increase in cells in S-phase. Graph bars represent mean of at least three experiments with standard error of the mean. Asterisks indicate the significant differences compared to the untreated sample (ctrl), this was tested using the non-parametric Mann-Whitney test. *p < 0.05, **p < 0.01, ***p < 0.001.

Having confirmed that HR is inhibited by HT in the used cell lines, we set out to determine the effects of PARP1 inhibition on the cytotoxicity of cDDP+HT treatment. To this end, clonogenic survival assays were conducted as schematically shown. Clonogenic assays were conducted to investigate the effect of the different treatments on cell survival Figure 2A. In Figure 2B, percentages of survival are normalized to the untreated samples. We observed a ∼50% decrease in cell survival after 1 h cDDP (5 μM) treatment alone. Hyperthermia as a monotherapy was less effective than treatment with a PARP1*-i*. Combining the PARP1-*i* with cDDP caused a slight decrease in cell survival, compared to samples treated with cDDP only. The combination of cDDP and HT was highly cytotoxic and killed > 85% of cells, while the combination of hyperthermia and PARP1-*i* was less effective. The triple treatment with cDDP, PARP1-*i* and HT resulted in a slightly lower cell survival than the double treatment of cDDP and hyperthermia, but this difference was not statistically significant.

To further explore the effects of various treatments, the induction of DSBs was quantified by counting γ-H2AX foci (Figure 2C). In R1 cells, cDDP treatment induced around 20 γ-H2AX foci, while in SiHa and HeLa a 3–4 fold more γ-H2AX foci were found 24 h after treatment with cDDP. Treatment with hyperthermia and cDDP induced a similar amount of DSBs (70–90) as the triple modality. The number of detected DSBs was relatively low after the single treatment with PARP1-*i* or hyperthermia. The combination of the PARP1-*i* with hyperthermia resulted in a slight increase in the number of DSBs in HeLa cells, but not in SiHa and R1 cells.

DNA content analysis was performed 48 h after the various treatments to test whether apoptosis is responsible for the observed induction of cell death (Figure 2D). All three cell lines showed > 20% of apoptotic cells after the single and double modalities, while after the triple treatment the induction of apoptosis increased significantly (up to 25–30%).

Cell cycle distribution was not significantly affected 16 h after treatments, except for the combination of hyperthermia and cDDP and the triple combination, which caused a ∼30–55% increase in accumulation of cells in S-phase (Figure 2E). These results indicate that addition of PARP1-*i* moderately sensitizes cells to the combinational cDDP+HT treatment, mainly by increasing apoptosis.

### Cell death and cell cycle arrest are prominent in cells treated with PARP1-*i*, cDDP and hyperthermia

**Figure 3:**
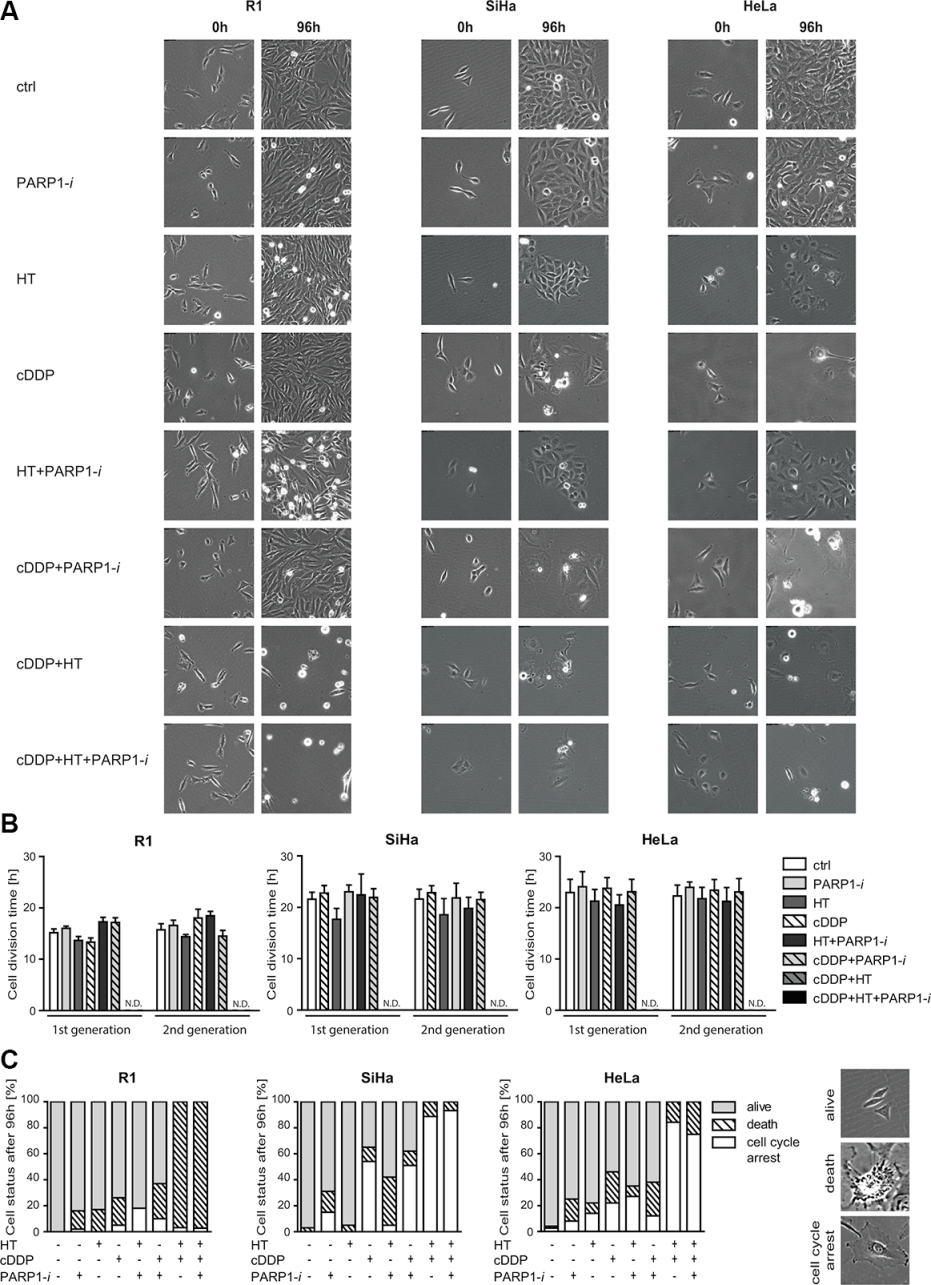
Time-lapse microscopy analysis 0 and 96 h after combination treatments with PARP1-*i* **(100 μM NU1025/continuously), HT (42°C/1 h), cDDP (5 μM/1 h)**. (**A**) Representative pictures of cells directly after treatment (0 h) and four days after treatment (96 h). (**B**) Cell division times of two generations post treatment. Cells that did not divide for over 50 h, but did not die, were regarded to be in ‘cell cycle arrest’. R1 cells have a shorter cell division time than SiHa and HeLa cells. But no significant differences were found after any of the treatments compared to the untreated cells. Graph bars represent mean with standard error of the mean of three independent experiments with the standard error of the mean of three independent experiments. (**C**) Graph bars with means represent cell status 96 h after treatment with examples pictures of each cell status on the right. Each condition has been performed in triplicate.

To study the cell cycle distribution in more detail, we followed cells using time-lapse microscopy for 96 h, starting immediately after the various treatments. In Figure 3A representative images of the cells are shown at 0 h (left column) and 96 h (right column) after the end of the indicated treatments. SiHa and HeLa cells ceased to grow rapidly after any treatment containing cDDP. However, thermochemotherapy alone (cDDP+HT) as well as the triple treatment were the most toxic for these cells (last two rows of pictures). The combination treatments had similar effects on the R1 cells, although they appeared to be less sensitive to cDDP alone. After 96 h, in cDDP+HT or cDDP+HT+PARP1-*i* treatments, most of the R1 cells died, while the SiHa and HeLa cells interrupted their cell cycle progression (Figure 3C). Cells that did not divide for over 50 h, but did not die, were regarded to be in ‘cell cycle arrest’. Nevertheless, the cell cycle time was unaffected in the surviving cells under the different treatment conditions, both in the first and second generation post-treatment, except for cells treated with cDDP+HT and the triple combination, where the few surviving cells were all in cell cycle arrest (Figure 3B).

### Adding PARP1-*i* to cDDP+HT treatment allows lowering cDDP dose while maintaining similar levels of cytotoxicity

**Figure 4:**
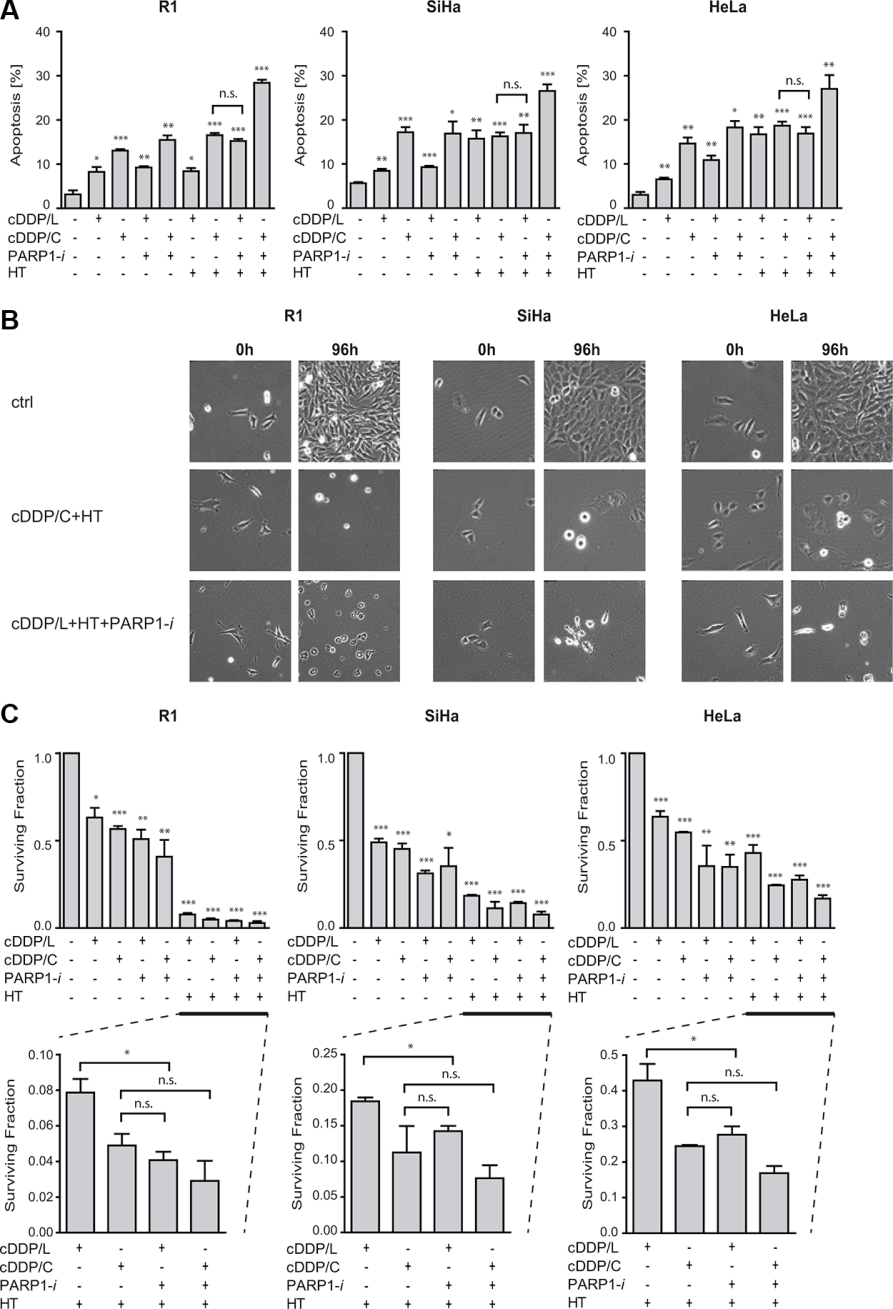
Addition of PARP1-*i* (100 μM NU1025/continuously) to the commonly used dose of cDDP (5 μM/1 h; cDDP/C) and HT (42°C/1 h) permits lowering of cDDP concentration (0.5 μM/1 h; cDDP/L) (**A**) To study apoptosis levels, the Nicoletti assay was performed 48 h after different treatments. No significant differences were observed between cDDP/L+HT+PARP1-*i* and cDDP/C+HT (R1: *p* = 0.10, SiHa: *p* = 0.86, HeLa: *p* = 0.22). (**B**) Time-lapse microscopy was performed 0–96 h after treatments. Pictures represent cells directly after treatment (0 h) and at the end of the analysis (96 h). (**C**) Clonogenic assays were conducted to study the effect 10-12 days after treatments. No significant differences were observed between cDDP/L+HT+PARP1-*i* and cDDP/C+HT (R1: *p* = 0.10, SiHa: *p* = 0.70, HeLa: *p* = 0.20). Graph bars show mean of at least three independent experiments with the standard error of the mean. Asterisks indicate the significant differences compared to the untreated sample (ctrl), tested using the non-parametric Mann-Whitney test. *p < 0.05, **p < 0.01, ***p < 0.001.

The effects PARP1-*i* could be masked by the relatively high cytotoxicity of the cDDP+HT combination treatment. To test this hypothesis, we compared the effectiveness of the commonly used dose of cDDP concentration (5 μM for 1 h; cDDP/C) to a tenfold lower cDDP concentration (0.5 μM for 1 h; cDDP/L). We found similar levels of cell death (Figure 4A) in cells treated with either cDDP/L+HT+PARP1-*i* or cDDP/C+HT (R1: *p* = 0.10; SiHa: *p* = 0.86; HeLa: *p* = 0.22). Time-lapse microscope analysis confirmed these results at 96 h after treatment (Figure 4B). Moreover, clonogenic survival analysis showed that cDDP/L+HT+PARP1-*i* had similar cytotoxic effects as the combination of cDDP/C+HT (Figure 4C). No significant differences were found in R1, SiHa and HeLa cells (*p* = 0.10, *p* = 0.70 and *p* = 0.20 respectively). These results demonstrate that using a PARP1-*i* could allow lowering cDDP dosage without significantly affecting the treatment efficacy. This might be of great importance for patients who do not tolerate the standard concentration of cDDP, due to toxicity.

## DISCUSSION

Our results show that the combination of a standard cDDP-dose plus HT already yields a high tumor kill. This agrees with clinical studies in women with recurrent cervical cancer [27]. Although, the addition of PARP1-*i* in an experimental setting only gives a minor enhancement of this effect, a moderate increase in cell death was observed in all cases. Moreover, at a ten-fold-lower cDDP concentration plus HT, the loss of tumorcidal effect can be compensated by PARP1 inhibition. These findings are consistent both in terms of cell surviving fraction, γ-H2AX foci staining and apoptosis. The advantage of the low-dose cDDP+HT+PARP1-*i* treatment, may be a lower cDDP related toxicity in patients. Our speculation would be that inhibition of PARP1-*i* does not cause an induction of DNA double strand breaks, but interferes with the b-NHEJ to prevent repair of double strand breaks [18, 19].

In the Netherlands, combinational treatment of cDDP and hyperthermia is one of the standard treatments options for women with recurrent cervical cancer after previous radiotherapy [14]. Previously irradiated tumors may have a lower perfusion caused by the irradiation inducing hypoxia, and hypoxic tumors are less sensitive to ionizing radiation [28–30]. Clinical trials have shown that HT is an effective chemosensitizer for cDDP [31, 32] that increases cDDP uptake in tumor cells and thus enhances the induction of DNA damage [33]. Additionally, as described earlier, hyperthermia also temporality inhibits one of the major DNA repair pathways, HR, by downregulation of BRCA2 [34, 35], explaining the rational for thermochemotherapy. However, one of the limiting effects of cDDP is nephrotoxicity.

Our results may have important clinical implications, as they suggest the possibility to diminish cDDP related toxicity, without reducing the desired cytotoxic effect. Therefore, combining a low dose of cDDP with HT and a PARP1-*i* may be a promising approach to increase tumor control whilst reducing systematic cDDP-toxicity, particularly in women with (recurrent) cervical cancer. The triple combination might be interesting for the treatment of other tumor types as well, because the treatment will also target tumors with wild-type *BRCA* status [36, 37] and it may also be an option for patients in whom radiotherapy is contra-indicated.

To assess if not only chemotherapy, but also hyperthermia dose can be reduced, it is also relevant to further elaborate whether reduction in time at 42**°**C or the use of a lower temperature combined with chemotherapy and a PARP1-*i* can cause comparable outcome. This is of clinical interest, since it is not always possible to achieve 42**°**C for 1 h at the tumor site. If the addition of a PARP1-*i* can be used to compensate for hyperthermia treatment at a shorter time or at a lower temperature, this may improve treatment outcome.

In several clinical trials the effect of high- and low-dose cDDP has been compared for squamous head and neck carcinoma [38–40] and cervical carcinoma [41], in which these studies report acceptable toxicity of low-dose cDDP in radiochemotherapy without significant differences in response rate between high- and low-dose cDDP.

Another way to decrease the severe nephrotoxicity caused by cDDP is to administer high-volume hydration in cDDP-based chemotherapy. This has been tested in clinical trials in patients with lung cancer [42, 43] and resulted in reduction of renal toxicity after high-volume hydration, while renal function was significantly worse after low-volume hydration. These effects might be related to osmolality, and both serum and urinary concentrations of cDDP [43, 44]. Likewise, as applying low-cDDP dose, a decrease in nephrotoxicity is achieved when administering high-volume hydration in patients.

Our results provide rational for adding PARP1-*i* to the standard cDDP+HT therapy, because this could slightly increase overall treatment efficacy, potentially allow reducing of cDDP concentration. It could also increase efficacy in tumor area that are poorly accessible for cDDP and it could increase efficacy in patient that are subjected to high-volume hydration. *In vivo* experiments need to be performed to confirm these findings.

## MATERIALS AND METHODS

### Cell lines

The rat rhabdomyosarcoma cells (R1) were grown in MEM and the cervical carcinoma cells (SiHa and HeLa) were grown in EMEM. The R1 cell line was created in our own laboratory [44]. SiHa and HeLa cell lines were obtained from the American Type Culture Collection (ATCC). All media contain 25 mM Hepes (Gibco-BRL life technologies, Breda, The Netherlands) supplemented with 10% heat-inactivated fetal bovine serum (FBS) and 2 mM glutamine. Cells were maintained at 37°C in an incubator with humidified air supplemented with respectively 2% and 5% CO_2_. The cell division time during exponential growth of R1 cells is approximately 16 h and of the cervical cancer cells 24 h.

### Chemical agents

Cells were treated with an optimized concentration [33, 45, 46] of 5 μM for 1 h cisplatin (cDDP; Platosin^®^, Pharmachemie B.V., Haarlem, The Netherlands). For experiments in which low and commonly used cisplatin concentrations were compared, cells were treated with either 0.5 μM (cDDP/L) or the standard dose of 5 μM (cDDP/C) for 1 h, respectively. The 0.5 μM dose was determined by performing a titration curve on clonogenic assays with concentrations between 0 and 5.0 μM cDDP dose combined with PARP1-*i* – and found that a 0.5 μM dose was the lowest dose of cDDP where the combination had an effect comparable to the effect of the standard concentration cDDP alone. The medium was refreshed directly after. To inhibit the b-NHEJ and SSB repair, 100 μM of PARP1-*i* (NU1025, Tocris Bioscience, Bristol, UK) was added 30 min prior to hyperthermia. After refreshing the medium, PARP1-*i* was re-added till the end of the experiment in the appropriate concentration.

### Hyperthermia

Incubation of the cells was performed by partially submerging the culture dishes in a thermostatically controlled water bath (Lauda aqualine AL12, Beun de Ronde, Abcoude, The Netherlands) for 1 h at 42°C. Temperature was checked in parallel dishes and the desired temperature (± 0.1°C) was reached in approximately 5 min. The atmosphere of the water bath was adjustable. R1 cells were heated in a 2% CO_2_/98% air atmosphere and SiHa and HeLa cells were heated in a 5% CO_2_/95% air atmosphere with an air inflow of 2 L/min.

### Immunohistochemistry

Cells were plated 24 h prior to treatment on sterile coverslips or 1.8 μm mylar membrane dishes. Cells on coverslips were treated with cDDP, PARP1-*i* and/or HT, while cells on mylar were treated with only hyperthermia and directly followed by irradiation with α-particles for 1 min. Next, cells were fixed with 2% paraformaldehyde after 15 min, 30 min (α-particles) or 24 h. After washing with PBS and incubated for 30 min with TNBS (PBS containing 0.1% Triton X-100 and 1% FCS), cells were stained with γ-H2AX (Millipore, dilution 1:100 in TNBS) and RAD51 antibody (dilution 1:25 in TNBS) as described by Aten *et al*. [47]. Finally, vectashield with DAPI (Life technologies, USA) was dropped at the slide and the coverslip is turned upside down on the slide, for scoring under the fluorescence microscope.

### Clonogenic assays

Cell survival was studied on R1, SiHa and HeLa cells using different treatment combinations; hyperthermia (1 h at 42°C), cDDP (cDDP/C: 5 μM for approximately 1 h, cDDP/L: 0.5 μM for approximately 1 h), PARP1-*i* (100 μM, continuously). Clonogenic assays were conducted as described by Franken et al. [48]. Cells were plated before treatment into 6-well culture plates (Costar, USA). Dishes were placed in an incubator with 5% CO_2_ at 37°C until sufficiently large clones were formed. Afterwards the medium is removed and cells were washed with PBS. A mixture of 6.0% glutaraldehyde (2–3 ml) and 0.5% crystal violet was added for at least 30 min at room temperature. Next, plates were washed with water and dried in normal air at room temperature. Colonies were counted under a light microscope [49]. Surviving fractions were calculated by dividing the plating efficiency of treated cells by that of control cells [50].

### Apoptosis assay

The Nicoletti assay [51] was used to study apoptosis in all cell lines. At 48 h after treatment cells were collected and pellets were resuspended in Nicoletti buffer (0.1% w/v Sodium citrate, 0.1% v/v Triton X-100 in demi water, pH 7.4). Analyses were made using flow cytometry (FACS Canto, BD Biosciences, USA).

### Cell cycle analysis

Cell cycle distribution was analyzed using the thymidine analogue 5-Bromo-2′-deoxy-uridine (BrdU, Sigma Aldrich, USA) that can incorporate into S-phase cells (60 min at 37**°**C). BrdU was added 16 h after treatment and 1 h prior to fixation in 2 ml PBS and 6 ml of 100% ethanol. The pellet was resuspended in pepsin-HCl (0.4 mg/ml, 0.1 N HCl) and cells were stored 30 min at room temperature. Cells were washed with PBT (0.5% Tween-20, Sigma Aldrich USA in 0.5 l PBS). The pellet was resuspended in HCl (2 N, Merck) and cells were incubated 30 min at 37**°**C. 100 μl primary antibody rat-anti-BrdU (Abcam, UK) in PBTb (1% bovine serum albumin, Sigma, in PBT) was added 1 h at 37°C. After washing, 100 μl IgG goat-anti-rat FITC (Abcam, UK) in PBTg (1% normal goat serum, Dako, USA, in PBT) was added and left for 1 h at 37°C. Final, PI (Sigma-Aldrich, USA) was added, cell suspensions were vortexed and directly measured using flow cytometry (FACS Canto, BD Biosciences, USA).

### Time-lapse microscopy

Cells were grown in 6-wells plates for 24 h, before treatments. Dishes were placed under a confocal microscope (Leica PS2, The Netherlands) with a temperature and CO_2_/air controlled chamber and cells were imaged for 96 h. A minimum of fifty cells were analyzed per condition.

### Statistics

All represented *in vitro* data are means ± standard error of the mean of at least three independent experiments. Cell survival and apoptosis were analyzed using SPSS (Chicago, IL, USA) statistical software using a non-parametric Mann-Whitney test.
